# Statistical Analysis of Surface Roughness, Burr Formation and Tool Wear in High Speed Micro Milling of Inconel 600 Alloy under Cryogenic, Wet and Dry Conditions

**DOI:** 10.3390/mi14010013

**Published:** 2022-12-21

**Authors:** Amjad Baig, Syed Husain Imran Jaffery, Muhammad Ali Khan, Mansoor Alruqi

**Affiliations:** 1 School of Mechanical and Manufacturing Engineering (SMME), National University of Sciences and Technology (NUST), Islamabad 44000, Pakistan; 2 Department of Mechanical Engineering, College of Electrical and Mechanical Engineering (CEME), National University of Sciences and Technology (NUST), Islamabad 44000, Pakistan; 3 Department of Mechanical Engineering, Shaqra University, Shaqra 11911, Saudi Arabia

**Keywords:** micro-milling, Inconel 600, renewable & sustainable manufacturing, green manufacturing, precision machining

## Abstract

Super alloys offer excellent mechanical and chemical properties at elevated temperatures that make them an attractive choice for aerospace, automotive and chemical processing, and marine applications. These alloys are, however, difficult to machine due to their high strength at elevated temperatures, low thermal conductivity and work hardening. In this study, micro milling of Inconel 600 super alloy has been carried out and the effects of the key input parameters (cutting speed, feed rate, depth of cut) on response parameters (burr formation, surface roughness and tool wear), under various cooling conditions (dry, wet and cryogenic), have been analyzed. High speed micro milling (range up to 80,000 RPM) was carried out, while keeping the feed rate values below and above the cutting edge radius. The Taguchi design of experiments was used during this study. The results have been analyzed using SEM and 3D optical microscopy. Analysis of Variance (ANOVA) revealed that the best surface roughness values can be achieved under cryogenic machining condition with an overall contribution ratio of 28.69%. It was also revealed that cryogenic cooling resulted in the highest tool life with the contribution ratio of cooling conditions at 26.52%.

## 1. Introduction

Demand of miniature products has increased manifolds over the last decade. The industrial sector, including chemical, aerospace, medical, microelectronics and automotive, has e become the main user of miniature finished products with high quality, high precision and high 3D accuracy [1,2,3,4,5]. While processes such as electro-discharge machining, laser micro manufacturing, lithography, and deep reactive ion etching can be used for micro components, these are not cost effective, and require highly skilled manpower and high operation time [6,7,8,9,10]. Micro milling is the most popular micro-machining process, as it is time and cost effective for the production of 3D miniature parts. The advancement in micro milling requires an in depth study of burr formations, surface roughness, micro cutting tool materials, micro tool coatings, work piece materials and process monitoring, to produce the quality product [11,12].

**Figure 1:**
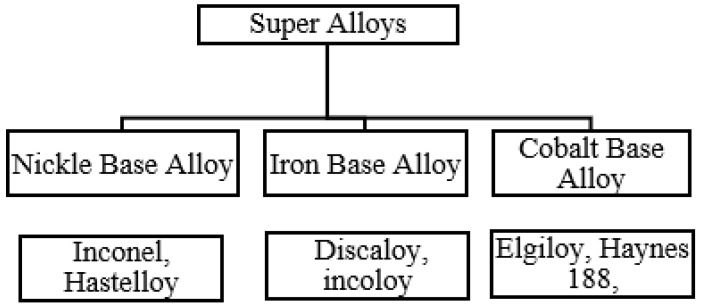
Super alloys classification.

The micro mechanical process is more economical than the other manufacturing systems in micro domain [13]. Many challenges are connected with micro-mechanical manufacturing systems requiring a standard change from macro manufacturing-processes. These problems are mostly due to the small size of tools, parts and processes. The quality micro tools are greatly affected by smaller vibrations and extreme forces, which is not good for the tools’ lives and for the tolerances of components. It is hard to notice the loss of the cutting edges of micro tools [14,15,16]. In the field of micro milling, the material properties, the tooling specifications and machining parameters play a vital role in controlling the products’ quality. The quality can be enhanced by controlling these input factors [17]. Super alloys are an attractive choice for the modern industry sector, due to their ability to retain their properties at extreme conditions. They are usually based on nickel chromium, cobalt, or nickel-iron. These super alloys are divided into three broad categories, namely Iron base, nickel base and cobalt base alloys, as shown in Figure 1. 

Most of the wrought nickel-base super alloys comprise at least 50% nickel and other materials, such as 10–20% Cr, 5–15% Co, up to 8% Ti and Al combined, and a small proportion of tungsten zirconium, boron, carbon niobium, molybdenum, and magnesium. Aluminum and chromium are required to improve surface stability [18]. Inconel 600 is an austenitic and stable solid-solution nickel–chromium-based super alloy having high strength and corrosion resistance at elevated temperatures; it also has excellent mechanical properties at temperatures in the range of −423 to 1300 °F [17,19,20,21,22]. The high temperature resistance makes Inconel 600 feasible for different applications involving temperatures from cryogenic to around 1095 °C. This alloy is widely used in the chemical industry, as well as aerospace industry. This alloy is used in the aeronautical field for making different engine and airframe components, i.e., exhaust liners, lock wires and turbine seals. 

Inconel 600 alloy shows poor machinability due to low values of thermal conductivity, work hardening, and a high affinity towards the cutting tool material [23]. High cutting temperature also causes deformation of the cutting tool [24]. The temperature at the cutting zones rises significantly during the cutting process, which decreases the tool life [25,26]. Other studies for nickel-based alloys reveal that high cutting speed is more effective for micromachining processes [27,28,29,30]. Tolerance and chatter issues arise due to lower elastic modulus [31]. In micro machining, the grain size effects the machining surface. As the tool moves from one grain to another grain, the tool causes the chip to break and deformation starts in the other grain, which causes the built-up edge formation. It is also found that when the feed/tooth is lower than the cutting-edge radius, the ploughing forces are increased. In micro-machining, due to the small depth of cut, there is a significant increase in friction between the material and the tool, which results in high temperatures and tool wear [32]. The burr formation in micro machining needs to be removed because post-processing cannot be applied to some parts. Burr formation is produced more in hard material due to the high tool wear. Poor edge and burr formation is more problematic in conventional machining. Post-processing is performed to avoid this problem. However, this is not possible for some parts in micro machining due to their small size [33]. The material properties become non-homogenous at micro level, thus the variation in hardness causes the tool to vibrate. This effect is greater at a low cutting speed and feed rate, which causes the irregular surface during the machining. A ductile material is easy to deform, which causes more burr and long chips. Burr formation is also significantly influenced by the tool run out.

Due to the environmental concerns associated with oil-based coolants, dry cutting has greatly been favored. Therefore, the selection of a coolant becomes more significant in order to improve overall efficiency during the machining of hard-to-cut super alloys. Green manufacturing is the use of environmentally friendly operations within the manufacturing field, in which fewer natural resources are used to reduce pollution and waste, recycle and reuse materials, and moderate emissions in their processes. Green manufacturing focuses on research to develop technologies and practices to lessen their impact on the environment. In this research, the use of liquid nitrogen is evaluated as an alternative to conventional cooling techniques. Conventional techniques, as reported by past researchers, such as flooded and minimum quantity lubrication, are known to be environmental as well as health and safety hazards. When compared with dry machining, cryogenic machining was found to improve tool life, as well as product quality in terms of burr formation and surface finish. The usage of coolant is more significant as there is a need for high speed machining [34]. The usage of coolant during machining processes is a well-known practice, ranging from High Pressure Coolant (HPC) [35,36] to minimum Quantity Lubrication (MQL) [37,38,39] and the subsequent introduction of cryogenic coolants [40,41,42,43,44], such as liquid nitrogen, Argon, etc. Liquid nitrogen is easier to handle and, therefore, a viable choice as a cryogenic medium. Coolant covers almost 20% of the total manufacturing cost [45,46] and, therefore, it needs to be weighed against the advantages. The use of coolant and the proper selection of machining parameters are highly important for productivity and the economy [42]. Another study also incorporated cryogenic cutting, in addition to dry cutting in the turning of Ti alloy [47]. The cutting speed and feed were analyzed during the use of cryogenic media. It was found that dual jet configuration was useful in achieving optimized values for tool wear, in addition to surface roughness. The motivation of this study was to investigate micro milling over a wide range of process parameters in the presence of cooling conditions, such as cryogenic, wet and dry, to cover the research gap and provide a set of proper combinations of process parameters.

## 2. Materials and Methods

Inconel 600 is a nickel–chromium based super alloy, possessing properties such as higher strength and hardness, with excellent corrosion resistance. Table 1 provides the mechanical properties of Inconel 600 and other common aerospace alloys, whereas Table 2 provides the chemical composition of the work piece (Inconel 600).

### 2.1. Experimental Set Up

**Figure 2:**
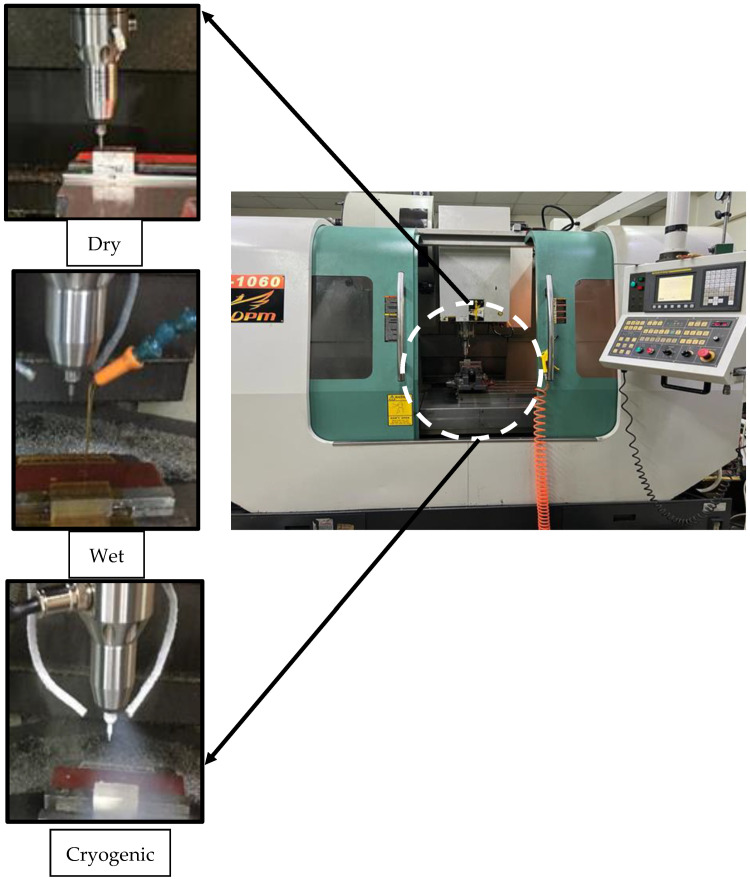
Experimental setup for dry, wet and cryogenic machining.

Micro milling experiments were performed using a computerized numerical control (CNC) Yida MV-1060 milling center. Cryogenic, wet and dry conditions were used during the experimentation phase by using the equipment shown in Figure 2. The cryogenic setup was installed in the CNC milling center with a high pressure cylinder XL-160 that can store 160 L of liquid nitrogen; this was used as a cryogenic media, as its effectiveness in the machining of super alloys and cutting tools had been reported in the literature [48]. It is the most widely-used media, due to its easy availability and inert nature [49]. A pressure of 20 psi was maintained through a pressure regulator which provided a flow rate of 4 L per minute. Two vacuum-insulated copper pipes with a 4 mm diameter were used to carry the cryogenic media through a bifurcated cryogenic needle valve. The usage of dual jets has been reported in the literature to produce optimum results [34,47,50]. The internal cooling system of the CNC milling center (Yida MV-1060) was used for the wet condition. The water-based coolant oil shell dromus B was used with a 6 L per minute steady flow rate. Table 3 shows the experimental conditions.

**Figure 3:**
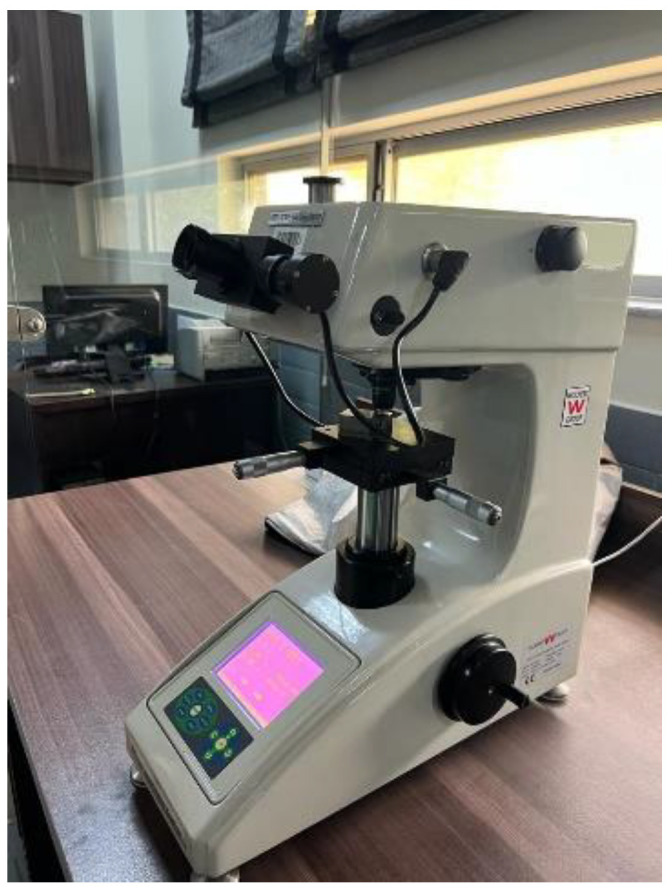
Micro-Vickers Hardness Tester.

A tool pre-setter (BMD Messwell 410V) was utilized for accurate measurements of the Z axis. The dimensions of the work piece were 10 mm × 20 mm × 50 mm. The surface roughness of the Inconel 600 alloy was analyzed using an Olympus DSX1000 digital microscope. The Micro-Vickers Hardness Tester (401MVD–WOLPERT GROUP) was used to find the hardness of the workpiece, as shown in Figure 3. The dwell time was 6 s and a 9800 mN force was used during these five tests, which were carried out at multiple locations on the work piece. The average value of micro hardness was calculated as 361 HV.

### 2.2. Cutting Tool Specifications

Initially, a carbide end milling tool, for levelling the work piece surface, was used and the finished surface was taken as a reference for micro milling experiments. Two flute ultra-fine tungsten carbide flat end mills, with a diameter of 500 µm, were used as micro-cutting tools during the current study. Table 4 shows the details/specifications of the micro cutting tools. The tool edge radius for each micro end mill tool was measured via scanning electron microscopy, and its average value was found to be 2.23 µm with a standard deviation of 0.16 µm. 

### 2.3. Design of Experiments

This research utilizes the Taguchi’s design of experiments for micro milling experiments. The Taguchi design of experiments [51], which is a popular method for designing experiments for research, was used to construct the orthogonal L9 array. The Taguchi method was preferred due to its efficiency in having lower numbers of runs required [52,53]. Taguchi orthogonal arrays are known to produce conclusive results [54]. Several past researchers have used it effectively [55,56,57,58]. Compared with the L9 array that can be used, to analyze 4 input parameters, each having three levels, a full factorial experimentation would require 81 experimental runs, as opposed to the fractional factorial approach of Taguchi that requires 9 experiments. Validation experiments were conducted to confirm the authenticity of the achieved result. In our particular case, 5 out of the 12 validation experiments were part of the original design of the experiment and were not required to be repeated. Only 7 experiments were repeated for validation. Even after including the verification experiments, the total number of experiments (16) stands at well below the 81 test runs required for a full factorial experimentation.

Key process parameters have been investigated to analyze their effect on response parameters, including tool wear, surface roughness, and burr intensity. These process parameters include (a) three levels of cutting speed, Vc (m/min); (b) three levels of feed rate, F (µm/tooth); (c) three levels of depth of cut, ap (µm); and (d) three cutting conditions including dry, wet and cryogenic. Details of key process parameters along with their levels are shown in Table 5.

Values for depth of cut (ap) were selected based on the recommended values from the literature. As per the Niagara Cutter (Cutter 2018), the depth of cut can be calculated as follows for micro tools having a diameter of 3.18 or below, as shown in Table 6.

Table 7 gives the details of L9 orthogonal array. The range of cutting speed (Vc) was selected to be from 75 m/min (48,000 RPM) to 125 m/min (80,000 RPM), which is based on values from the literature review; this is because different researchers have reported the speed variation to be between 16 m/min and 141 m/min [59,60,61]. The value of feed rate (F) was selected at equal to, below and above the cutting edge radius to see its effect on the response parameters in the micro milling of Inconel 600 alloy. The range for the undeformed chip thickness, in terms of feed rate values, was selected to cover values both above and below the edge radius at 1 micron per tooth (half the edge radius), 2 micron per tooth (equal to the edge radius) and 4 micron per tooth (two times the edge radius).

### 2.4. Responses

It includes tool wear, surface roughness, top burr width for ‘up milling and down milling cases’, and top burr height ‘up and down milling cases’. To ensure accuracy, the various readings of each response parameter were calculated, and the average values of the results have been recorded and shown in Table 7.

### 2.5. Burr Formation Measurement

**Figure 4:**
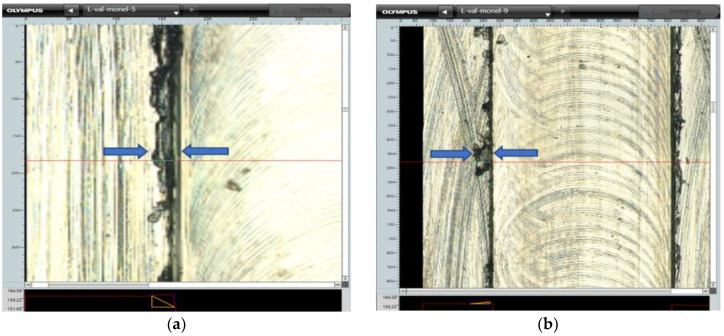
(**a**) Top burr width measurement; (**b**) Top burr height measurement.

The scanning electron microscope, SEM–JEOL JSM-6490A, and Olympus DSX 1000 digital microscope at multiple magnifications, have been used for analyzing and calculating the top burr width and top burr height in both cases, namely the up milling and down milling sides. The average values of the results have been calculated (Figure 4) and displayed in Table 7.

**Figure 5:**
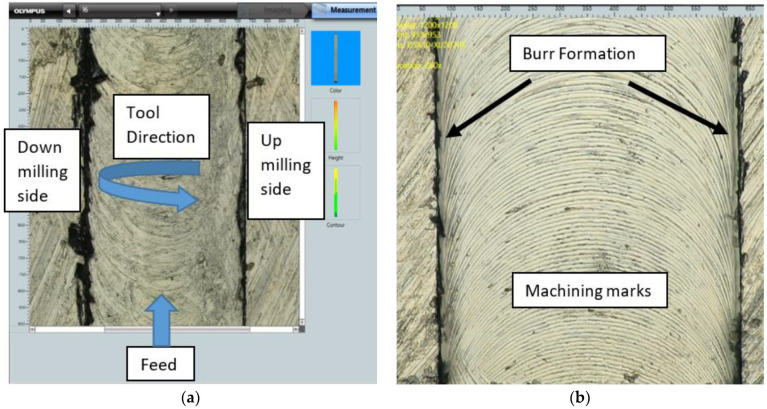
(**a**) Up and down milling sides; (**b**) Burr and machining marks on machined surface.

Figure 5a shows the micro milled slot of Inconel 600 alloy, indicating the feed direction, rotation of the micro tool, and up and down milling sides. Meanwhile, Figure 5b indicates the edges of the micro milled slot of the work piece, showing dull black and uneven protrusions, which are burr-produced during micro machining operations. It also indicates that the dull black marks (away from the edges of the milled slot) are actually machining marks on the finished surface, and therefore these can be neglected.

### 2.6. Surface Roughness

SEM, optical microscopy and analytical technique was used for the calculation of surface roughness of all micro milled slots. It provides the opportunity to identify the micro surface textures resulting from micro milling operations. Multiple locations were selected on the finished surface of the work piece for calculations of surface roughness. Table 7 shows the average value of results. 

### 2.7. Tool Wear

**Figure 6:**
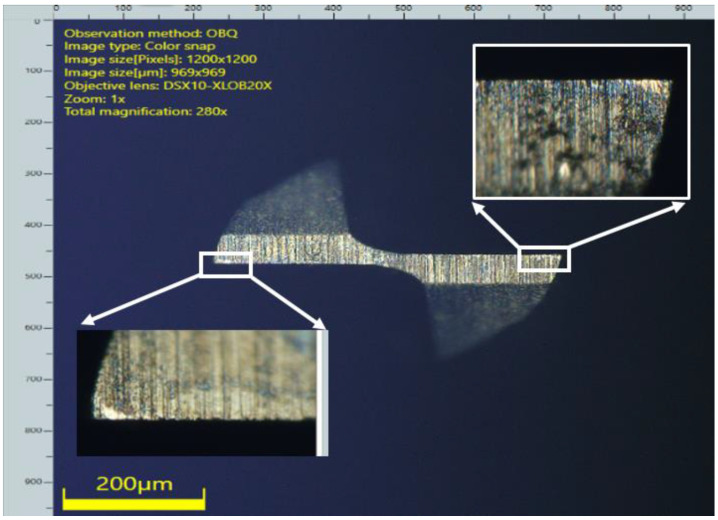
Tool wear.

Tool wear plays a key role in industrial machining, as the quality of the finished product and dimensional accuracy are influenced by it [49]. Therefore, tool wear was also given due consideration in this study. Optical microscopy, along with SEM and statistical techniques, were used for calculation and analysis purposes. Figure 6 indicates the effect of input machining parameters on tool wear. ANOVA (Analysis of Variance) was carried out for tool wear by using the same methodology that was used for surface roughness. Flank face tool wear progression was measured from the magnified images that were taken near the cutting tool edge radius of micro tools, before and after the machining process. The average values of results have been recorded in Table 7.

## 3. Results and Discussions

Each experiment was performed twice to measure the response parameter. Table 7 shows the outcome of these experiments. Statistical technique (ANOVA) was utilized for analysis and plotting the results of each response parameter. It is important to highlight here that even small errors and amounts of noise greatly affect the value of output responses in micro machining processes, due to their increased sensitivity. Jaffery et al. found that residual dual effects were more significant in the micro milling of metals, compared to the macro machining domain [62]. Variations in the initial and subsequent run were also recorded; these variations are mainly due to variation in human error, tools quality and machine noise.

### 3.1. Application of ANOVA 

Analysis of variance (ANOVA) is a technique which shows the significance of key process parameters on the output responses. ANOVA utilizes multiple equations for calculating the significance of individual parameter as output responses. Equation (1) gives the sequence of sum squares (SSA) for each parameter, where *A* indicates process parameter, *n* shows the number of runs at a particular level, *i* is the level, *T* indicates the response value at each run, and *N* shows the total number of runs. Equation (2) computes the total sum of squares (SST), and Equation (3) gives the sequential sum of squares of error (SSe). The percentage contribution of individual parameters can be calculated with the help of Equation (4). The parameter with the higher F–ratio value indicates its high impact on output responses and vice versa, whereas the *p*-value indicates the probability that a test would fail. Table 8, Table 9, Table 10, Table 11, Table 12 and Table 13 show the analysis of the variance for surface roughness, burr width (up and down milling cases), burr height (up and down milling cases) and tool wear.

(1) $$\;SS_{A\;}=\overset{3\;}{\underset{i=1}{\sum }}\frac{A_{i}^{2}}{n}-\frac{{\left(\sum_{j=1}^{N}\;\;T_{j}\right)}^{2}}{N}\;$$

(2) $$\;SS_{T\;}=\overset{N}{\underset{j=1}{\sum }}\;T_{j}^{2}-\frac{{\left(\sum_{j=1}^{N}\;T_{j}\right)}^{2}}{N}\;$$

(3) $$\;SS_{e\;}=SS_{T\;}-\overset{Z}{\underset{i=A}{\sum }}\;\;SS_{i\;}\;\;$$

(4) $$\;\%CR{\;}_{=}\frac{SS-(df\;XMSS_{Res})}{SS_{T\;}}\times 100$$

### 3.2. Surface Roughness

**Figure 7:**
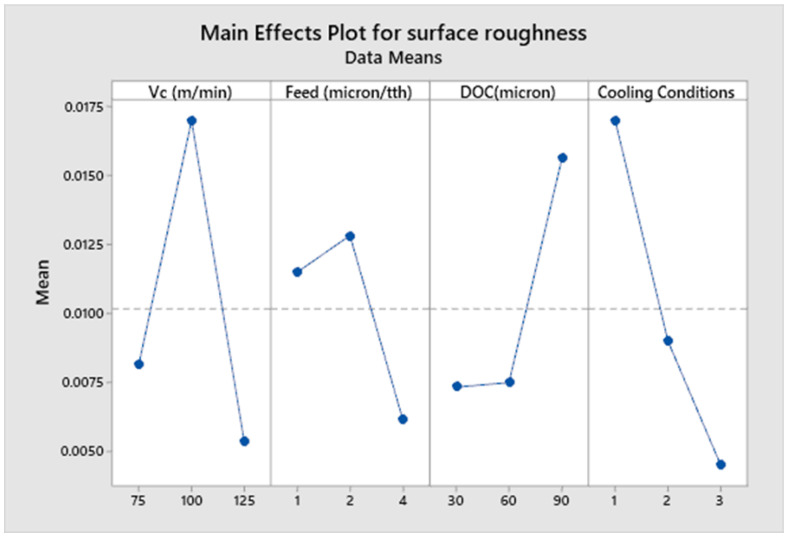
Main effects plot for surface roughness of Inconel 600.

It is a vital output response parameter because the quality of the final product is directly related to the finished surface of the final product. This study shows that surface roughness is influenced by multiple cooling conditions, along with other parameters such as feed rate, cutting speed and depth of cut. Figure 7 shows the plotted results of surface roughness against different process parameters.

The plotted results in Figure 7 show that the cryogenic cooling condition helped to produce the lowest value of surface roughness, followed by wet conditions which gave the next highest result. Dry conditions showed the highest value of surface roughness. It is because of this fact that better surface roughness is achieved with coolant, as it serves as a lubricant between the sliding surfaces [63]. Another reason is the substantial alteration of the coefficient of friction between the work piece and tool interaction during the use of coolant [64]. The other fact is that, under dry conditions (no-coolant/lubricant), a higher value in the tool wear also increases the surface roughness. Comparable findings have also been reported by previous researchers [43,65]. The phenomena of coolant penetration have also been reported by various researchers [41,66,67]. Therefore, all of the above-mentioned facts contribute towards the better surface finish under cryogenic and wet conditions. The ANOVA Table 8 shows that cooling conditions we found to be the most significant factor, with a contribution ratio of 28.69%. The main effects plot (Figure 7) shows that higher values of surface roughness are achieved as the depth of cut increases. When the depth of cut increases, the cutting forces, in addition to the tool vibration, contribute towards higher values of surface roughness in the Inconel 600 alloy. When the value of depth of cut increases from 30 µm to 90 µm, the value of surface roughness also increases, which might be due to micro tool vibrations and the cutting forces involved. The outcome from the experimentation shows that the best surface finish is achieved when feed rate is selected to be just above the cutting edge radius. The value of the micro cutting tool edge radius was found to be 2.23 µm. This indicates the sensitive relationship between the fee rate and micro cutting tool edge radius in achieving the best quality of finished surface. The cutting tool edge radius has direct relation to feed rate, as the cutting edge radius is influenced by undeformed chip thickness, resulting in the variation of values of surface roughness. It can also be concluded that micro machining carried out at a feed rate below the cutting edge radius, does not give significant advantage over the feed rate selected above the cutting tool edge radius. We see that a feed rate selected above the value of cutting edge radius gave the best surface roughness value. High speed micro milling was considered because high cutting speed during the experimentation phase avoids built-up edges (BUE), which are the result of a low cutting speed. Low cutting speed produce BUE, which include chatter and a worsening of the surface roughness. This phenomenon further deteriorates the surface quality of the workpiece due to BUE. The welded chips, which are result of BUE, are removed from the finished surface, resulting in surface roughness.

The ANOVA in Table 8 shows that the cutting speed Vc is the next highest significant factor, with a contribution ratio of 26.50% out of total variability. The main effects plot (Figure 7) shows that the influence of cutting speed on surface roughness at various speed values is nonlinear. The surface roughness increases initially near transition phase, as the cutting speed increases from 75 m/min to 100 m/min, while it starts decreasing in the high-speed range, as the cutting speed increases from 75 m/min to 125 m/min (80,000 RPM), thus improving the quality of finished surface.

### 3.3. Burr Formation

Deburring of finished products in the micro machining domain is extremely difficult. In many cases, post processing for deburring of micro components may not be possible. In the micro domain, these processes may result in distortion of the work piece, a change in the dimensional accuracy, and influence mechanical properties, such as surface finish, residual stresses, elastic limit and fatigue strength. Measuring 3D burr shapes in the micro domain is a challenging task, as there is no common approach available for their measurement. Previous studies show that burr can either be compared qualitatively as per their shapes, or it can be measured in term of burr width and burr height [68,69,70,71]. Burr height and width in combination do not reveal the fact that they can be curled in shapes. However, burr height and width can be measured in terms of assessment of burr overall formation. Therefore, in this study, top burr width and top burr height have been considered to show an overall trend in the field of micro milling domain. Statistical technique, optical microscopy and SEM have been used to measure the top burr width and height, and the corresponding results are displayed in Table 7. Burr formation on up milling and down milling sides were analyzed, and top burr width and top burr height were measured using an Olympus DSX 1000 digital microscope at multiple magnifications. These parameters were investigated over their entire range by keeping the feed rate value below and above the cutting edge radius value. It is also highlighted here that the intensity of the burr formation towards the down milling side is greater in comparison to the up milling side. Moreover, burr size (including width and height) formed at the down milling side is greater in comparison to the up milling side. Localized cutting velocity explains this phenomenon. A localized region on the down milling side has a local cutting velocity that always remained less than the localized cutting velocity on up milling side. This results in the formation of a larger burr on the down milling side. Previous researchers have also reported the same behavior while investigating the burr formation behavior during micro milling of Ti-alloy (Ti-6Al-4V) [72]. This is another reason that might explain the higher intensity of the formation of burr on the down milling side, in terms of frictional forces regarding the micro tool rubbing against the burr and might caught in the flute of the micro end mill.

**Figure 8:**
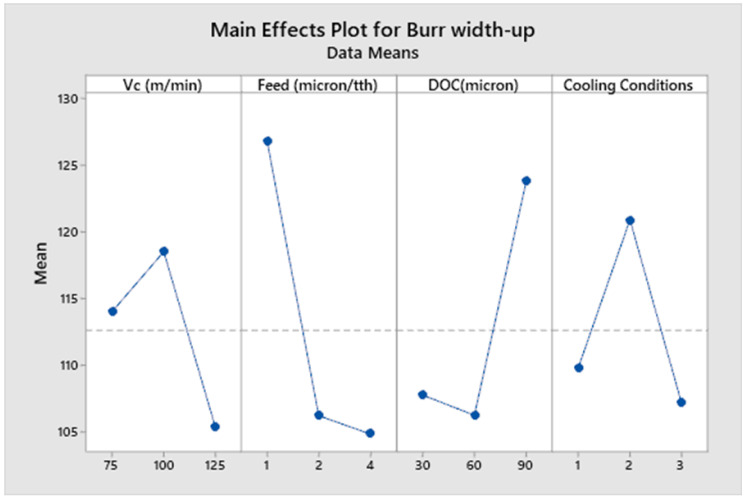
Main effects plot for burr width up milling case.

**Figure 9:**
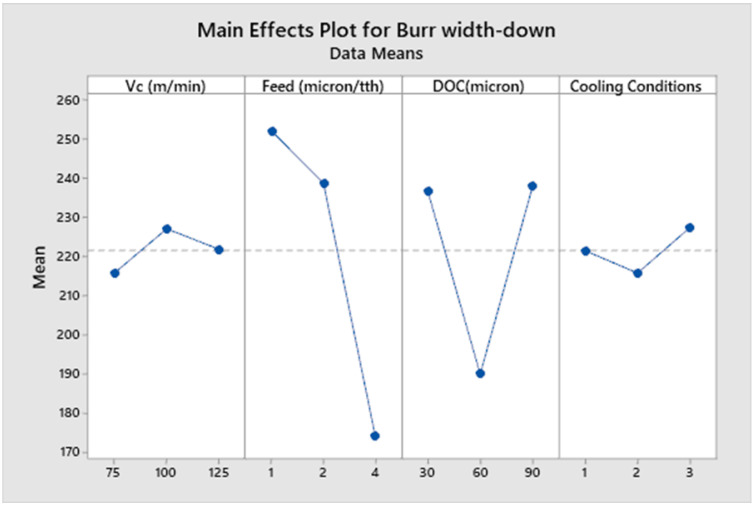
Main effects plot for burr width down milling case.

**Figure 10:**
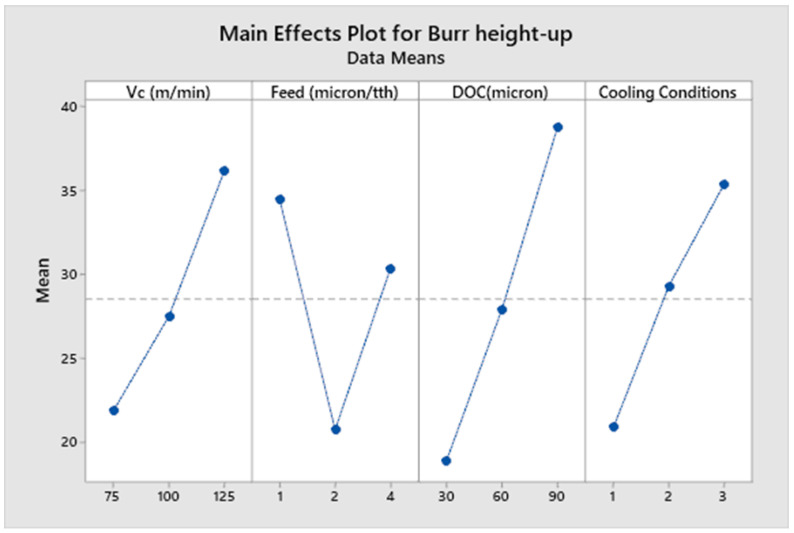
Main effects plot for burr height up milling case.

**Figure 11:**
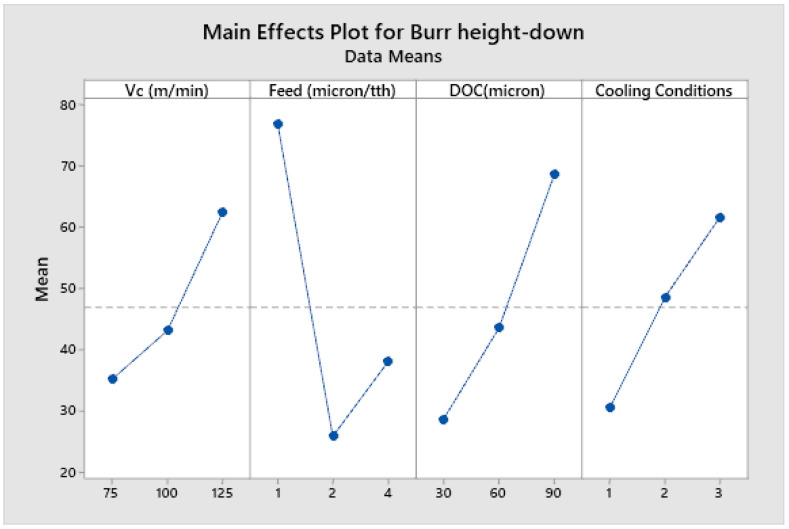
Main effects plot for burr height down milling case.

Figure 8, Figure 9, Figure 10 and Figure 11 show the main effect plots related to top burr width (up and down milling cases) and burr height (up and down milling cases), and the results are shown in Table 7. We observe the nonlinear behavior of different parameters, as shown in the main effect plots for burr formation. Considering the main effect plots of Burr width (up and down milling), it is clear that micro machining below the cutting edge radius does not have a significant advantage in comparison to machining above the cutting tool edge radius. This phenomena has also been reported by Jaffery et al. in his investigation into the micro milling of Ti-6Al-4V alloy [62]. The ploughing effect below cutting edge is more prominent in micro milling, so when the feed is selected to be above the cutting edge radius minimum, the value of burr width is achieved. Cutting speed and cooling conditions are non-significant factors, as shown in ANOVA Table 9 and Table 10. The burr width in both cases (up and down milling) initially increases at a lower cutting speed but it drops at a higher cutting speed range. Figure 10 and Figure 11 show the main effect plots of burr height for both cases (up and down milling). It is observed from these plots that minimum burr height is achieved at a feed rate of 2 µm/tooth, close to the cutting edge radius (2.23 µm). Their values start increasing when the feed rate crosses the range from below cutting edge radius to above cutting edge radius. Their values also increase linearly with the increase in the value of the depth of cut. The burr height values also increase as the value of the cutting speed increases.

Table 9, Table 10, Table 11 and Table 12 show the ANOVA (Analysis of Variance) for each process parameter on the burr width and height (both up and down milling cases), along with their contribution ratios. Table 9 and Table 10 show that feed is the most significant factor for top burr width (up and down milling cases), with contribution ratios of 34.70% (burr width up milling case) and 66.21% (burr width down milling case), respectively. The next significant factor is the depth of cut, with contribution ratios of 21.7% and 28.77%, respectively. The cutting speed and cooling conditions appear to be non-significant factors for burr width in both cases. Table 11 and Table 12 show the analysis of variance for burr height up and down milling cases, respectively. The depth of cut is the most significant factor for the burr height up milling case, with a contribution ratio of 29.51%; meanwhile, the feed rate is the most significant factor for the burr height down milling side, with a contribution ratio of 43.88%. Burr height has a direct correlation to the depth of cut, as its value increases with the increase in the value of depth of cut. Minimum burr height is achieved at a feed rate close to the cutting edge radius and a with a combination of process parameters: a cutting speed of (Vc) 75 m/min, a feed rate of 2 µm/min, a depth of cut of 30 µm, and dry cooling conditions.

### 3.4. Tool Wear Analysis

**Figure 12:**
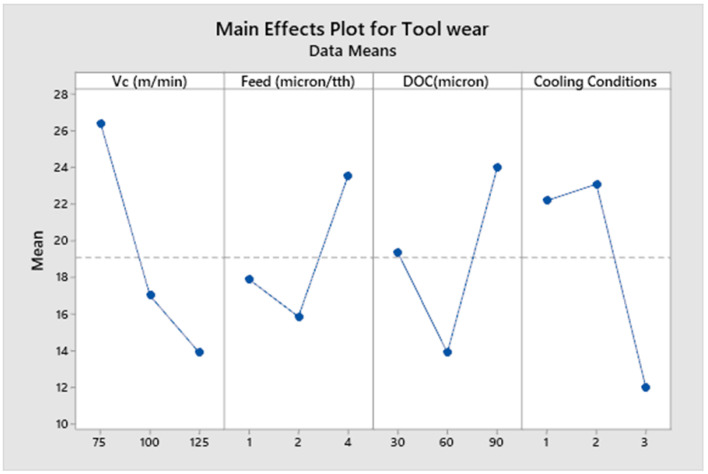
Main effects plot for tool wear.

There is a direct interaction between tool and work piece, therefore, the tool wear plays a vital role in the quality of the finished product. The desire for a high surface finish and accuracy in micro components, further increases its importance in the micro machining domain. In this study, tool wear was investigated to find the proper combination of process parameters to minimize the tool wear while working on super alloys such as Inconel 600. The results are shown in Table 7. Figure 12 shows the main effects plot for the tool wear of micro tools under various machining parameters. Since maximum tool wear was considered for the tool flank wear and flank wear, lands for both the bottom edge and the side edge merge at the corner of the end mill, and both bottom edge flank wear and side edge flank wear show identical values.

Figure 12 shows that the micro tool operating at a cutting speed of Vc = 125 m/min, a feed rate of F = 2 µm/tooth, and depth of cut of ap = 60 µm under cryogenic condition, shows the longest tool life. Meanwhile, the micro tool operating at Vc = 75 m/min, F = 4.0 µm/tooth and a depth of cut of ap = 90 µm, showed a higher material removal rate with a shorter tool life. In addition to the above mentioned parameters, burr formation and the surface finish of the product are also important parameters when gauging the micro tool performance. It is noted from the main effect plot (Figure 12), that tool wear varies non linearly, as the feed rate changes from 1.0 µm/tooth to 4.0 µm/tooth, with a pounced effect at a feed rate equal to 2.0 µm/tooth; this is close to the average cutting tool edge radius of micro tools (2.23 µm). This might be due to the elastic recovery effects which are more intense when a micro tool is cutting at a feed rate comparable in value to the minimum chip thickness. Cryogenic cooling conditions show the best value for tool wear, which might be due to the substantial alteration to the coefficient of friction between the work piece and the micro tool interaction during the use of liquid nitrogen. In the machining of aerospace alloys that have a low thermal conductivity, the chip flows over the rake face of the tool up to a certain length, consisting of seizure and slip regions, known as the tool chip contact zone. As a result, considerable heat is generated and passed on to the tool since the thermal conductivity of the chip is low; this is shown in Table 1. This contrasts with the thermal conductivity of the tool material, tungsten carbide, which is around 40 W/mK [73]. Under cryogenic cooling conditions, the thermal gradient across the chip causes the chip to warp and lose contact. It also becomes brittle and disengages from the tool, thereby reducing heat generation in the seizure zone. Furthermore, the low temperature also improves tool life by reducing its thermal softening and deterioration due to high temperature abrasion.

Table 13 shows the analysis of variance for tool wear. Process parameters, including cutting speed and cooling conditions, are significant with contribution ratios of 29.70% and 26.52%, respectively. Delicate micro tools are more prone to noise factors or uncontrollable changes in environments, including heat in the cutting zone. This not only adds to residual effects, but also contributes towards micro tool wear. Cryogenic cooling conditions played a vital role in reducing the micro tool wear, as shown in the main effects plot (Figure 12) and ANOVA Table 13. Liquid nitrogen media maintains the temperature at −196°C, which enhances tool life while preventing the production and transfer of heat to the micro tool. It also helps in extricating heat from the cutting zone. Cryogenic media also reduces the contact length between the tool and chip, which also contributes towards the reduction of frictional heat generation. The same phenomena has also been reported in the literature [42].

## 4. Validation Experiments

During this study, the significant process parameters under various cooling conditions were taken into consideration and their effects on response parameters, including surface roughness, burr formation and tool wear, were investigated. The Taguchi design of experiments and statistical technique ANOVA were used to identify the contribution of process parameters, based on ‘the smaller is the better model’. The results displayed in Table 7 were as predicted by the Taguchi methodology. Later on, validation experiments were performed using the best and worst combinations of process parameters to confirm the validity of the achieved results. Confirmatory test results are shown in Table 14, and these are comparable with the results obtained via the Taguchi analysis. The trends already predicted using the Taguchi design of experiments were confirmed via the validation experiments. The optimum conditions produced the best results, compared to the initial results.

## 5. Conclusions

The current study investigated the effects of key machining parameters, under multiple cooling conditions, on surface roughness, burr formation and tool wear, during high speed micro milling of Inconel 600 alloy. The following conclusions can be drawn based on the achieved results:

- ‘Cooling condition’ was found to be the most significant factor with a contribution ratio of 28.69% towards surface roughness, followed by the cutting speed at 26.50%.
- Cutting speed had the highest contribution towards tool wear at 29.70%, followed by cooling media at 26.52%.
- Cryogenic cooling conditions produced the lowest value for tool wear at 10.49 µm, resulting in increased tool life, as confirmed in the validation results (tool wear 9.81 µm).
- Higher values of cutting speed, at 125 m/min, produced better surface roughness, with the minimum value of depth of cut at 30 µm, due to the lubricating effect the use of coolant had, improving the surface roughness.
- Feed rate was found to be the parameter with the most significant effect on burr formation in both cases, i.e., up and down milling case, with the following contribution ratios:
  ◦ Burr width- down milling case   66.21%
  ◦ Burr height down milling case   43.88%
  ◦ Burr width-up milling case      34.70%
- Depth of cut was the most significant factor for burr height (up milling case), with a contribution ratio of 29.51%. The next significant factors towards burr formation with contribution ratios for both cases (up and down milling) were as follows:
  ◦ Burr width down milling case   28.26%
  ◦ Burr width-up milling case     21.77%
- A feed rate of 4 µm/tooth was selected above the cutting edge radius and gave the best surface finish, in comparison to work with a feed rate of 2 µm, below the cutting edge radius.
- The down milling side experienced the greater intensity of burr formation when compared to the up milling side, due to the larger localized velocity effect on the down milling side.
- Minimum burr width was achieved at a feed rate of 4 µm, above the cutting edge radius, while the minimum burr height was achieved at a feed rate of 2 µm, close to the cutting edge radius of 2.23 µm.
- An increased depth of cut at 90 µm results in poor surface finish, due to higher vibrations and cutting forces in the cutting zone of micro milling operations.

## Figures and Tables

**Table 1:** Mechanical properties of aerospace alloys.

	Inconel 600	Monel 400	Inconel 718	Ti-6Al-4V
Density (gcm^−3^)	8.4	8.8	8.2	4.5
Hardness (HB)	360	110–150	390	320
Tensile strength (MPa)	1050	512–620	1600	950
Elastic modulus (GPa)	205	179	205	113.8
% Elongation	25–30	48	15	14
Thermal conductivity (W m^−1^ K^−1^)	10	21.8	11.4	6.7

**Table 2:** Inconel 600 chemical composition (wt%).

Ni	Cr	Mn	Si	Fe	S	C
72	16	1.0	0.5	8.0	0.015	0.15

**Table 3:** Experimental conditions.

Work Piece Material	Inconel 600
Cutting length (mm)	10 mm
Cutting conditions	Dry wet and crogenic
Milling type	Full immersion
Tool diameter (mm)	0.5
Number of Flutes	2

**Table 4:** Details of cutting tool with specifications.

Detail	Information
Brand	North Carbide Tools
Type	End mill
Material	Tungsten carbide
Diameter (mm)	0.5
Number of flutes	2
Rockwell hardness (HRC)	60
Overall length (mm)	50
Helix angle (°)	35
Blade length (mm)	1
Cobalt content (%)	12

**Table 5:** Process parameters.

Parameters	Units	Level 1	Level 2	Level 3
Cutting Speed (Vc)	m/min	75	100	125
Feed Rate (F)	µm/tooth	1	2	4
Depth of cut (ap)	µm	30	60	90
Cutting conditions	-	Dry	Wet	Cryogenic

**Table 6:** Process parameters.

Parameters	Formula	Value	Remarks
Depth of cut (ap)	Dia of tool × (0.25 to 0.05)	-	Cutter diameter = 0.5 mm (500 micron)
Minimum ap	0.5 × 0.05	0.025 µm	
Maximum ap	0.5 × 0.25	125 µm	

**Table 7:** L9 orthogonal array with process parameters and corresponding response parameters.

	Input Parameters				Response Parameters					
Test	Cutting Speed Vc (m/min)	Feed (F) (µm/tooth)	DoC (ap) (µm)	Cutting Conditions	Surface Rough-ness (Ra-µm)	Burr Width Up Milling (µm)	Burr Width Down Milling (µm)	Burr Height Up Milling (µm)	Burr Height Down Milling (µm)	Tool Wear (µm)
1	75	1	30	dry	0.0135	120.533	261.3435	10.4875	30.4205	28.562
2	100	1	60	wet	0.0145	134.5785	220.2745	33.629	71.3025	14.6005
3	125	1	90	cryogenic	0.0065	125.272	274.506	59.267	128.8035	10.49
4	75	2	60	cryogenic	0.0025	95.7775	207.222	20.372	25.5075	10.879
5	100	2	90	dry	0.032	120.506	260.458	22.3805	27.374	21.75
6	125	2	30	wet	0.004	102.37	248.327	19.4915	24.718	14.914
7	75	4	90	wet	0.0085	125.713	178.7195	34.799	49.6515	39.67
8	100	4	30	cryogenic	0.0045	100.408	200.459	26.5255	30.6545	14.6165
9	125	4	60	dry	0.0055	88.3915	117.629	29.7705	33.7165	16.2065

**Table 8:** Analysis of variance in surface roughness.

Source	DF	Seq SS	Adj SS	Adj MS	F-Value	*p*-Value	Contribution
Vc (m/min)	2	0.000444	0.000444	0.000222	6.07	0.021	26.50%
Feed (micron/tth)	2	0.000149	0.000149	0.000075	2.04	0.186	8.91%
DOC (micron)	2	0.000272	0.000272	0.000136	3.72	0.066	16.24%
Cooling Conditions	2	0.000481	0.000481	0.000241	6.57	0.017	28.69%
Error	9	0.000330	0.000330	0.000037			19.65%
Total	17	0.001677					100.00%

**Table 9:** Analysis of variance burr width up milling.

Source	DF	Seq SS	Adj SS	Adj MS	F-Value	*p*-Value	Contribution
Vc (m/min)	2	536.4	536.4	268.2	2.19	0.168	10.26%
Feed (micron/tth)	2	1814.8	1814.8	907.4	7.40	0.013	34.70%
DOC (micron)	2	1138.7	1138.7	569.3	4.64	0.041	21.77%
Cooling Conditions	2	636.8	636.8	318.4	2.60	0.129	12.17%
Error	9	1103.9	1103.9	122.7			21.10%
Total	17	5230.6					100.00%

**Table 10:** Analysis of variance burr width down milling.

Source	DF	Seq SS	Adj SS	Adj MS	F-Value	*p*-Value	Contribution
Vc (m/min)	2	383.9	383.9	191.9	1.80	0.220	1.21%
Feed (micron/tth)	2	20,939.3	20,939.3	10,469.7	98.36	0.000	66.21%
DOC (micron)	2	8938.4	8938.4	4469.2	41.98	0.000	28.26%
Cooling Conditions	2	405.3	405.3	202.6	1.90	0.204	1.28%
Error	9	958.0	958.0	106.4			3.03%
Total	17	31,624.9					100.00%

**Table 11:** Analysis of variance for burr height up milling.

Source	DF	Seq SS	Adj SS	Adj MS	F-Value	*p*-Value	Contribution
Vc (m/min)	2	621.9	621.9	310.9	2.76	0.116	15.28%
Feed (micron/tth)	2	594.6	594.6	297.3	2.64	0.126	14.61%
DOC (micron)	2	1200.9	1200.9	600.5	5.32	0.030	29.51%
Cooling Conditions	2	637.0	637.0	318.5	2.82	0.112	15.65%
Error	9	1015.4	1015.4	112.8			24.95%
Total	17	4069.8					100.00%

**Table 12:** Analysis of variance for burr height down milling.

Source	DF	Seq SS	Adj SS	Adj MS	F-Value	*p*-Value	Contribution
Vc (m/min)	2	2352.3	2352.3	1176.16	15.40	0.001	12.13%
Feed (micron/tth)	2	8508.1	8508.1	4254.05	55.69	0.000	43.88%
DOC (micron)	2	4906.7	4906.7	2453.35	32.12	0.000	25.30%
Cooling Conditions	2	2935.8	2935.8	1467.90	19.22	0.001	15.14%
Error	9	687.4	687.4	76.38			3.55%
Total	17	19,390.4					100.00%

**Table 13:** Analysis of variance tool wear.

Source	DF	Seq SS	Adj SS	Adj MS	F-Value	*p*-Value	Contribution
Vc (m/min)	2	508.0	508.0	253.99	8.95	0.007	29.70%
Feed (micron/tth)	2	188.4	188.4	94.18	3.32	0.083	11.01%
DOC (micron)	2	305.2	305.2	152.62	5.38	0.029	17.84%
Cooling Conditions	2	453.7	453.7	226.84	8.00	0.010	26.52%
Error	9	255.3	255.3	28.36			14.92%
Total	17	1710.5					100.00%

**Table 14:** Results of validation tests.

Test	Cutting Speed (Vc) (m/min)	Feed (F) (µm/ tooth)	DOC (ap) µm	Cooling Conditions	Type	Output Parameters	Run 1	Run 2	Average
1	125	4	30	cryogenic	Best	Surface roughness	0.001	0.0032	0.0021
2	100	2	90	dry	Worst	Surface roughness	0.038	0.026	0.032
3	125	4	60	dry	Best	Burr width up milling	100.955	75.828	88.3915
4	100	1	90	wet	Worst	Burr width up milling	146.276	185.724	166.00
5	75	4	60	wet	Best	Burr width down milling	116.230	101.578	108.904
6	100	1	90	cryogenic	Worst	Burr width down milling	399.588	458.236	428.912
7	75	2	30	dry	Best	Burr height up milling	10.426	7.454	8.94
8	125	1	90	cryogenic	Worst	Burr height up milling	72.813	45.721	59.267
9	75	2	30	dry	Best	Burr height down milling	30.582	10.294	20.438
10	125	1	90	cryogenic	Worst	Burr height down milling	136.953	120.654	128.804
11	125	2	60	cryogenic	Best	Tool wear	14.204	5.428	9.816
12	75	4	90	wet	Worst	Tool wear	39.998	39.342	39.67

## Data Availability

The data of this research are being used for further extended research and can be made available in due course.
